# HTLV-1-Associated Myelopathy (HAM) Incidence in Asymptomatic Carriers and Intermediate Syndrome (IS) Patients

**DOI:** 10.3390/pathogens13050403

**Published:** 2024-05-13

**Authors:** Rosa Maria do Nascimento Marcusso, Tatiane Assone, Michel E. Haziot, Jerusa Smid, Victor A. Folgosi, Carolina Rosadas, Jorge Casseb, Augusto C. Penalva de Oliveira, the HTLV-1 Clinical Definition Working Group

**Affiliations:** 1 Instituto de Infectologia Emílio Ribas, São Paulo 01246-000, Brazil; rmmarcusso@gmail.com (R.M.d.N.M.); michelhaziot@gmail.com (M.E.H.); jsmid77@hotmail.com (J.S.); rdcassia@uolcom.br (A.C.P.d.O.); 2 Departamento de Medicina Legal, Bioética, Medicina do Trabalho e Medicina Física e Reabilitação, Faculdade de Medicina, Universidade de São Paulo, São Paulo 01246-000, Brazil; tatianeassone@usp.br; 3 Departamento de Dermatologia, Faculdade de Medicina, Universidade de São Paulo, São Paulo 01246-000, Brazil; victor.angelo@usp.br; 4 Section of Virology, Department of Infectious Disease, Imperial College London, London SW7 2BX, UK; c.rosadas-de-oliveira@imperial.ac.uk

**Keywords:** HTLV-1-associated myelopathy, incidence, Brazil, surrogate markers, early stage

## Abstract

Several studies suggest that HTLV-1 infection may be associated with a wider spectrum of neurological and clinical manifestations that do not meet diagnostic criteria for HAM. These conditions may later progress to HAM or constitute an intermediate clinical form: intermediate syndrome (IS), a mid-point between asymptomatic HTLV-1 carriers and those with full myelopathy. Thus, we determined the incidence of HAM cases in the HTLV-1-asymptomatic and IS patients, and the clinical/laboratory associated markers. A total of 204 HTLV-1-positive patients were included in this study, divided into two groups: Group 1, including 145 asymptomatic HTLV-1 subjects (ASY), and Group 2, including 59 patients with inflammatory clinical symptoms in more than three systems and a high proviral load (PVL). During a 60-month follow-up time, with the age ranging from 47 to 79 years, ten patients of the fifty-nine initially diagnosed as IS developed HAM (iHAM), and two patients of the initial 145 ASY developed HAM directly. Women were more prevalent in all groups. For the iHAM patients, the age ranged from 20 to 72 years, with a mean of 53 (±15 SD). Older age was associated with the development of HAM, higher PVL and IS; however, there was no any specific symptom or clinical sign, that was associated with risk for iHAM. In conclusion, IS cases could be an early phase of development of HAM. These findings show the presence of higher incidence probabilities in our cohort than previously reported.

## 1. Introduction

HTLV-1 was the first human retrovirus described in 1981, and is the causative agent of Adult T Leukemia (ATLL) and HTLV-1-associated myelopathy (HAM) [1]. Until recently it was believed that only a few individuals who carry the virus (3–5%) developed classical syndromes (ATLL and HAM), or exhibit inflammatory clinical evidence of systemic HTLV-1 infection, such as uveitis, dermatitis, arthritis, pneumonitis, among others, as well as subclinical neurological disturbances [2,3,4]. Furthermore, a meta-analysis has shown it is associated with increased overall mortality not explained by HAM and ATLL [5]. There is a consensus that a pro-inflammatory microenvironment is the hallmark of the immunological profile of HAM patients and may be responsible for the inflammation and degeneration in the spinal cord [6].

Indeed, clinicians observed several minor signals and symptoms associated with HTLV-1 in asymptomatic carriers during the follow-up, and we have described a non-myelopathic neurological dysfunction, designated Intermediate Inflammatory Syndrome (IS), which does not fulfill Castro-Costa diagnostic criteria of HAM [3,4]. Another important point in this context is that, on average, an individual with HTLV-1 takes seven years to obtain a diagnosis of HAM. Usually, they begin follow-up in non-specialized HTLV service, and the majority of the cases already have advanced stages of the disease, leaving little alternative for successful treatment [6]. 

There are few reports in the literature of the incident HAM. For example, in the Caribbean Basin, the incidence rate is 1.2 [7], and in Martinique during 14 years of follow-up presented a decline from 2001 to 2005 and from 2006 to 2010, with an HAM incidence rate of 4.27 and 2.03, respectively [8]. In Brazil, it was reported that the incidence density was 5.3 cases/1000 cases/year [9]. 

Indeed, to identify iHAM individuals is a challenge, since the majority cases of HAM in our cohort spend about eight years to have a HTLV-1 diagnosis after HAM development, because there are a lack about the knowledge on HTLV into health professionals. Thus, the early identification of this cases is very important for better options for treatment. This present report determined the incidence of HAM cases in the HTLV-1-asymptomatic and IS patients, and the clinical/laboratory associated markers.

## 2. Methods

### 2.1. Cohort Characteristics, Patient Recruitment, and Sampling Strategy

The HTLV outpatient clinic of the Institute of Infectious Diseases Emilio Ribas, São Paulo City, Brazil, has enrolled 727 individuals living with HTLV-1. This open cohort was created in 1997, and has been the place for some important studies and those regarding the characterization of IS [3]. This study was conducted during the period from January 2015 to January 2020.

All volunteers underwent serological screening for HTLV-1 at the Emilio Ribas Institute of Infectious Diseases (IIER), utilizing GOLD ELISA HTLV-1/2 (Diasorin, Dartford, UK), followed by confirmation with Western Blot (HTLV Blot 2.4^®^, MP Diagnostics, Copenhagen, Denmark) and in-house nested PCR, a criteria to entry in the IIER HTLV Cohort [10]. Blood samples were collected in K3-EDTA (0.054 mL/tube) and plasma was obtained by centrifugation (15 min, 2500 rpm). Peripheral blood mononuclear cells (PBMCs) were separated by Ficoll-hypaque density gradient centrifugation (GE Healthcare Life, Austin, TX, USA). Cells were washed with saline solution, and cells were stored, as “dry pellet” at −80 °C. DNA was extracted from 1 × 10^6^ PBMCs using a commercial kit (Illustra Tissue and Cells Genomic Prep Mini Spin kit, Fairfield, CA, USA), according to the manufacturer’s instructions, and stored at −80 °C for HTLV-1 proviral load quantification.

### 2.2. Data Collection and Clinical Classification

Clinical and laboratorial data were entry into the electronic database RedCap^®^ 1.0 was carried out by two administrative assistants, verified by the first and last author for quality control, and updated regularly during the follow-up with the clinicians. All clinical data from our cohort have been updated regularly over the last 22 years. 

A total of 449 individuals living with HTLV-1 who did not meet Castro-Costa criteria [11] for HAM were identified in the HTLV-1 Out Clinic, during the prospective study. Patients co-infected with HIV and/or HCV, those with ATL, and those who did not attend the clinic (were lost to follow-up for unknown reasons, and the team was unable to make contact) within the last two years, were excluded from this study (n = 204). The remaining 143 asymptomatic subjects were included and were seen at least once a year. A clinical evaluation and a standardized screening neurological examination were performed by MH (a board-certified neurologist and blinded for HTLV-1 clinical condition) for all subjects. To investigate intermediate syndrome (IS), the clinicians used an anamnesis form (Supplemental Material), where they examine eye, urogenital, rheumatic, oral, dysautonomia, and skin clinical manifestations not correlated with possible other base causes. Those patients, who, during follow-up developed symptoms which fulfilled the HAM diagnostic criteria recommended by an international consortium were classified as incident HAM (iHAM) [3].

### 2.3. Quantification of HTLV-1 Proviral Load (PVL)

The amount of HTLV-1 provirus in samples was quantified using real-time PCR, using primers and probes targeting the HTLV-1 *pol* gene, normalized against the human albumin reference gene. Samples were tested in duplicate to ensure precision, and results were reported as HTLV-1 DNA copies per 10^6^ PBMCs [12]. All laboratory analysis was done at the Institute of Tropical Medicine of São Paulo University (IMTSP/USP). The results of HTLV-1 proviral load (PVL), were obtained through the Hospital Information and Management System (SIGH) and were included in the RedCap database previously.

### 2.4. Statistical Analysis

For demographic variables and laboratories, the odds ratios (OR) with their 95% confidence intervals were calculated. The *p*-value < 0.05 was considered statistically significant. Proportions of variables transformed in categoricals were compared by Fisher’s exact test. The Kruskal–Wallis test, chi-square test, and hazard ratio (HR) were performed using the statistical package IBM SPSS Statistics software v26.0 (SPSS).

### 2.5. Ethical Issues

The study was approved by Emilio Ribas Institute of Infectious Diseases ethical board under protocol 86379218.6.1001.0061. Signed informed consent was obtained from all participants.

## 3. Results

**Figure 1:**
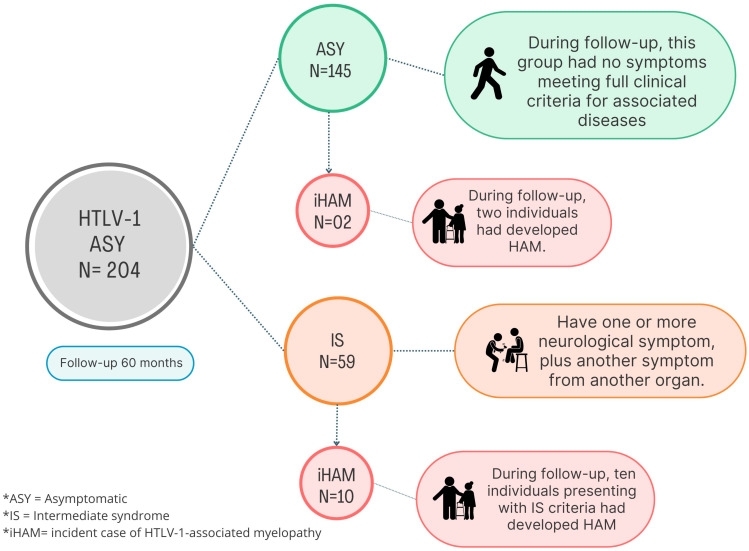
Algorithm used in this study to evaluate the incidence of HAM.

**Figure 2:**
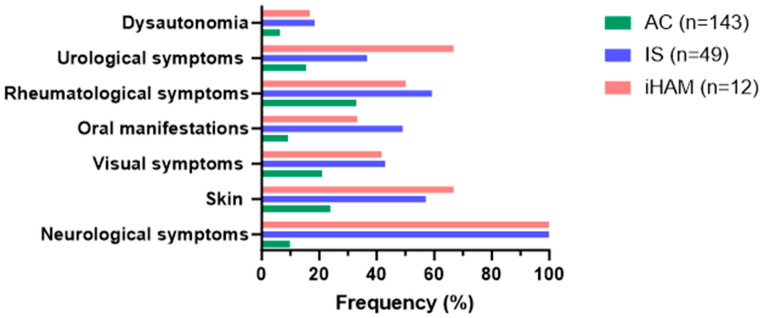
Clinical symptoms by group. AC: asymptomatic carriers; IS: intermediate syndrome; iHAM: incident HTLV-1-associated myelopathy.

A total of 204 individuals living with HTLV-1 were recruited for this study, divided into two groups; group 1 comprised 145 (71.1%) asymptomatic HTLV-1 subjects (ASY), and group 2 comprised 59 (28.9%) patients diagnosed with IS (Figure 1). Women were more numerous among the asymptomatic, corresponding to 79.6% in the IS group and 66.7% in the iHAM group (Table 1). During a 60-month follow-up, 5.9% (12/204) of those living with HTLV-1 developed HAM (iHAM). Incident HAM was more frequent amongst individuals classified as IS at baseline. A total of 10/59 (16.9%) patients initially diagnosed as IS developed HAM, whilst only 2/145 (1.4%) patients initially classified as ASY developed HAM during follow-up. The most frequent age group was that of 55 to 60 years; for iHAM, patient age ranged from 47 to 79 years, with a mean of 64 (±9 s.d.) and a median of 57 years. Age was associated with the development of HAM. The LPA was higher in iHAM compared to IS patients (*p* = 0.020), and the most frequent clinical symptoms in the iHAM cases were in the urologic system (66.7%) and in the skin (66.7%) (Table 1, Figure 2).

The analysis regarding the incidence of clinical symptoms (Table 1), showed that all IS cases presented neurological symptoms as expected, and 57.1% presented skin symptoms. On the other hand, 100% of iHAM cases presented neurological symptoms and 66.7% had skin and urological symptoms. It is important to highlight that 83.3% of iHAM cases went through the IS stage before evolving into HAM. Table 2 showed a hazard ratio (HR) indicating a risk of 173.2 for iHAM in the IS group compared with the ASY group, at a 5-year follow-up.

## 4. Discussion

In this retrospective study, it was observed that over a five-year follow-up time, from 2015 to 2020, 59 intermediate syndrome (IS) cases, diagnosed according to guidelines issued in a previous report [3], were diagnosed in our cohort in Sao Paulo city, Brazil. Of these 59 patients initially diagnosed as IS [3], ten (20.4%) developed full-blown HAM over five years of follow-up, and two (1.4%) cases of iHAM were directly diagnosed from ASY subjects [11]. The incidence of HAM in this subset was striking, indicating that it may be higher than previously described in Brazil and elsewhere [2,13]. In Salvador, it was observed during 8 years that 30% of the asymptomatic subjects developed any neurological signal or symptom associated with HTLV-1-infection [4]. Those findings are similar to previous studies and may be explained by the fact that the Sao Paulo cohort includes a high percentage of North and Northeast immigrants [14]. Thus, the identification of a set of clinical manifestations present in individuals with HTLV-1 (IS group) could translate the inflammatory nature of the disease in early years of the infection and correlate with the risk of converting from asymptomatic to HAM and clinical myelopathy outcome. 

A Martinique cohort with a 14-year clinical follow-up, including 123 patients with HAM, reported a rapid decline of HAM incidence from 2001 on in comparison to the 1986–2000 period, which could be explained by regional characteristics [2,8]. In contrast, a UK cohort of HAM patients from 1993 to 2007 including 48 patients (79.2% female, mean age 46 years), reported that the median time spanned from the onset of symptoms to diagnosis and to the last follow-up was 2 and 11.6 years, respectively. The most common first recalled symptom was unilateral leg weakness, supporting the present results.

Even though the laboratory biomarkers were not the major aim of this study, it was possible to observe that iHAM progression [15,16], was also observed for the higher DNA HTLV-1 proviral load. Some studies have shown that patients who developed HAM during follow-up had high proviral load levels (PVL) (> 1%) before the onset of disease, but there were no PVL increases with time, similar to findings in other studies from Brazil [17]. The data suggest that there is a trend toward reaching an equilibrium plateau of PVL over time, which is an individual characteristic [18]. A significant rate of ASY subjects sustain high PVL levels for a long time without developing clinical symptoms associated with HTLV-1 infection, so serial quantification of PVL does not seem to be a good prognostic marker for HAM [19]. 

HAM is an inflammatory condition that affects the individual in different organs, such as sphincter dysfunction, mucous membranes, skin, and other auto-immune diseases [4,20,21,22,23,24,25]. In fact, the majority of iHAM cases presented few of these clinical sphincter signals, and it could be explored whether this predicts the risk of HAM development into the asymptomatic cases. Furthermore, 90% of the incident cases were women, similarly found elsewhere, and women showed a strikingly higher risk for HAM development, similar to another study in the UK [19].

For those recent-onset HAM cases, it should be important to check for laboratory markers and to evaluate the clinical response, using neurological scales in a long fashion. Taken together, these new approaches have the potential to generate insights, which can influence treatment [26], improving the quality of life and guiding new clinical endpoints. Five years may not be enough time to address the incidence of HAM, and a larger sample size and longer follow-up should improve the confidence in the observations painted in this study. However, based on our results a prediction of the risk for the development of full-blown HAM cases can be made, and it points to higher probabilities than previously reported. So, we emphasize that asymptomatic patients should be evaluated at least every six months, using more strict clinical trials.

## Figures and Tables

**Table 1:** Clinical and proviral load evolution from asymptomatic or intermediate syndrome (IS) to HAM in five years.

	ASY (n = 143)	IS (n = 49)	iHAM (n = 12)	Total (n = 204)	*p* Value
Age					
Mean ± SD	50.0 ± 14.5	50.6 ± 14.6	64.2 ± 9.6	51.0 ± 14.6	0.004 *
Median	50	52	64	52	
Gender					
Male	39 (27.3%)	10 (20.4%)	4 (33.3%)	53 (26.0%)	0.535 **
Female	104 (72.7)	39 (79.6%)	8 (66.7%)	151 (74.0%)	
Neurological symptoms	14 (9.8%)	49 (100%)	12 (100%)	75 (36.8%)	≤0.001 **
Skin	34 (23.8%)	28 (57.1%)	8 (66.7%)	70 (34.3%)	≤0.001 **
Visual symptoms	30 (21.0%)	21 (42.9%)	5 (41.7%)	56 (27.5%)	≤0.001 **
Oral manifestations	13 (9.1%)	24 (49.0%)	4 (33.3%)	41 (20.1%)	≤0.001 **
Rheumatological symptoms	47 (32.9%)	29 (59.2%)	6 (50.0%)	82 (40.2%)	0.004 **
Urological symptoms	22 (15.4%)	18 (36.7%)	8 (66.7%)	48 (23.5%)	≤0.001 **
Dysautonomia	9 (6.3%)	9 (18.4%)	2 (16.7%)	20 (9.8%)	0.035 **
PVL					
Mean ± SD	44.5 ± 143.3	323.9 ± 354.7	1635.6 ± 299.7	205.2 ± 820.7	≤0.001 *
Median	10	178	65.5	29.5	
Evolution					
ASY⇒IS	-----	49 (100%)	-----	49 (24.0%)	
ASY⇒IS⇒HAM	-----	-----	10 (83.3%)	10 (4.9%)	0.004
ASY⇒HAM	-----	-----	2 (16.7%)	2 (1.0%)	

* Kruskal–Wallis test; ** chi-square test. Age: *p* _ASY-IS_ = 0.675, *p* _ASY-Iham_ = 0.001, and *p* _IS-iHAM_ = 0.001. PVL: *p* _ASY-IS_ = 0.778, *p* _ASY-iHAM_ = 0.001, and *p* _IS-iHAM_ = 0.003. PVL: proviral load; SD: standard deviation; ASY: asymptomatic; IS: intermediate syndrome; and iHAM: incident HAM.

**Table 2:** Incidence of HTLV-1-associated myelopathy (HAM) among those with diagnosis of intermediate syndrome (IS).

iHAM	ASY (n = 145)	IS (n = 59)	HR	95% CI	*p* Value
Yes	2 (1.4%)	10 (16.9%)	173.2	[36.3−826.8]	≤0.001
No	143 (98.6%)	49 (83.1%)			

## Data Availability

The data presented in this study are available on request from the corresponding author due to legal and ethical reasons.

## Supplementary Materials

The following supporting information can be downloaded at: https://www.mdpi.com/article/10.3390/pathogens13050403/s1, File S1: Anamnesis Form to SI investigation.
