# Supplementary material for: HTLV-1-Associated Myelopathy (HAM) Incidence in Asymptomatic Carriers and Intermediate Syndrome (IS) Patients
Source: Pathogens. 2024 May 13;13(5):403. doi: 10.3390/pathogens13050403 (PMC11124065; doi:10.3390/pathogens13050403)
Supplement: Supplementary file 1 [file pathogens-13-00403-s001.zip › pathogens-2964637-supplementary.pdf]

USP NAP RETROVÍRUS
NÚCLEO DE APOIO À PESQUISA

Anamnesis Intermediate Syndrome

General Information

Date of Evaluation:________________ Perfomed by:_______________

Personal Data

Name:____

Date of Birth:________________ Hospital ID:_______________

e-mail:__________________________ Phone : (   ) _______________

1. EYE MANIFESTATIONS

Xerophthalmia:
○ Yes ○ No

Previous uveitis without established cause:
○ Yes ○ No

Schirmer test: ___________ mm

2. UROGENITAL MANIFESTATIONS

Urological symptoms:
○ Yes ○ No

If yes, check the symptom bellow:
○ Dysuria
○ Nocturia
○ Urgency
○ Incontinence
○ Urge-incontinence
○ Hesitation

Erectile Dysfunction: ○ No ○ Mild ○ Moderate ○ Severe

Urodynamic Disturbances:
○ Hyperreflexia Hyperreflexia
○ Hyperreflexia with detrusor dyssinegism
○ Pure hypocontractile vesico-sphincteric
○ Areflexia
○ Others: ____

Prolonged use of Foley catheter: ○ Yes ○ No
Repeat infection urinary tract: ○ Yes ○ No
Urinary tract ultrasound alterations: ○ Yes ○ No

3. RHEUMATIC MANIFESTATIONS

Have any rheumatic manifestations

- ○ Yes ○ No

If yes, check the symptom bellow:

- ○ Arthralgia > 2 joints
- ○ > 3 months or Arthritis
- ○ Myositis with creatinofosfoquinase (CPK) alteration
- ○ Spondylitis

Known Rheumatological Disease: (excludes)

- ○ Rheumatoid Arthritis
- ○ Mixed connective tissue disease (MCTD)
- ○ Systemic lupus erythematosus
- ○ Polymyositis
- ○ Fibromyalgia
- ○ Osteopenia
- ○ Osteoporosis
- ○ Myositis with CPK alteration
- ○ Spondylitis
- ○ Other: ____

4. ORAL MANIFESTATIONS

Have any Oral manifestations:

- ○ Yes ○ No

If yes, check the symptom bellow:

- ○ Xerostomia
- ○ Periodontal disease
- ○ Erythematous
- ○ Candidiasis
- ○ Others:_________________ ______________________________

Sialography with salivary gland hypofunction: ○ Yes ○No ○ Not performed

5. DYSAUTONOMY

Have any signal of dysautonomy:

- ○ Yes ○ No

If yes, check the symptom bellow:

- ○ Postural hypotension
- ○ Erectile Dysfunction
- ○ Vasovagal syncope
- ○ Others:_________________ ______________________________

6. SKIN MANIFESTATIONS

Have any skin manifestations (no systemic disease or known cause):

○ Yes ○ No

If yes, check the symptom bellow:

○ Xerodermia

○ Acquired ichthyosis

○ Infectious dermatitis

○ Seborrheic dermatitis

CHECK THE XERODERMIA AREA BELLOW

Male:

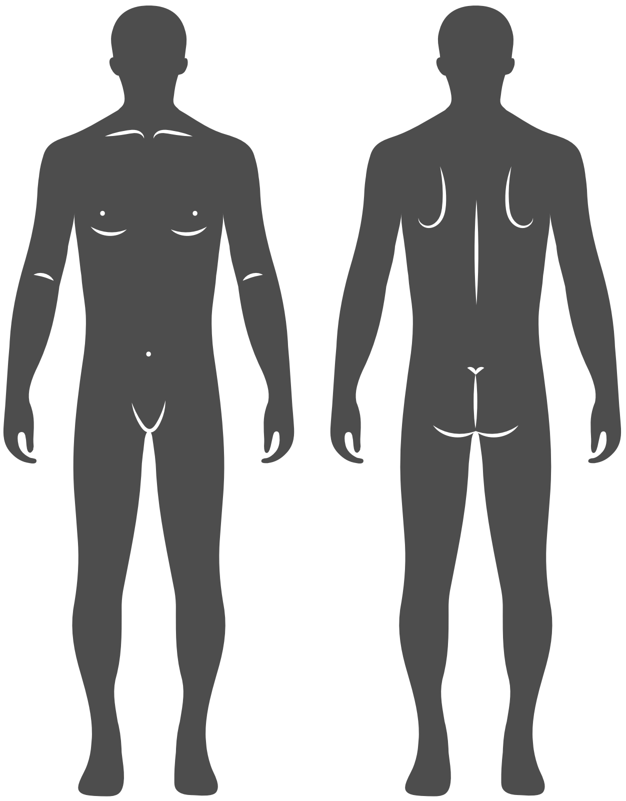

Female:

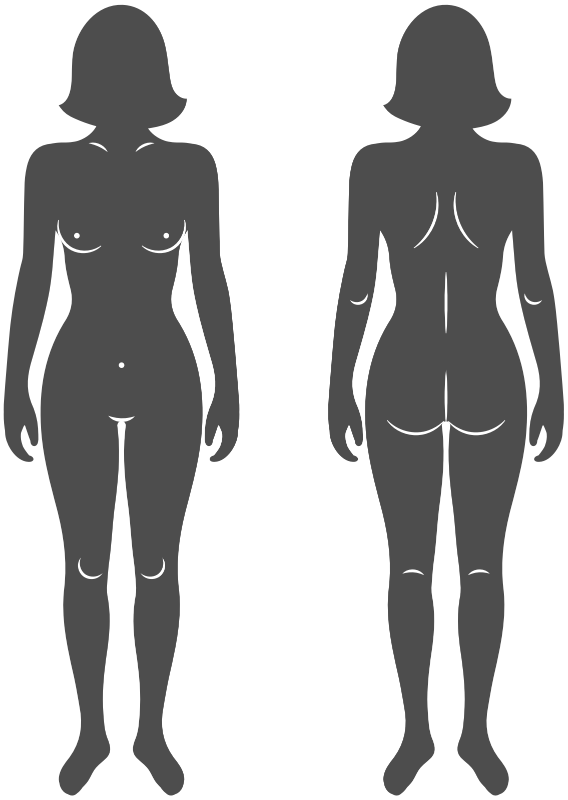

7. Peripheral Neurological Manifestations

Have any Peripheral Neurological Manifestations manifestations ?

- ○ Yes ○ No

If yes, check the symptom bellow:

- ○ Strength in upper and lower limbs,
- ○ Cranial nerves function
- ○ Patellar
- ○ Biceps
- ○ Plantar reflexes
- ○ Appraisal of the vibration sense

Have any presence of minimal changes in muscular strength or gait?

- ○ Yes ○ No

The patiente are able to:

- ○ Yes ○ No

To walk on their heels

- ○ Yes ○ No

To walk on toes

- ○ Yes ○ No

To walk on tandem gait

- ○ Yes ○ No

To rise from a chair without help from their arms

- ○ Yes ○ No

8. Adittional Comment
